# Cardiac Resynchronization Therapy in Children

**DOI:** 10.2174/157340309787048167

**Published:** 2009-01

**Authors:** Anjan S Batra, Seshadri Balaji

**Affiliations:** 1 Departments of Pediatric Cardiology, Children’s Hospital of Orange County, University of California, Irvine, California; 2 Departments of Pediatric Cardiology, Oregon Health and Science University, Portland, Oregon

## Abstract

Cardiac Resynchronization therapy has become an important management tool in adults with heart failure and dilated cardiomyopathy. The role of CRT in children with CHF is still unclear. Evidence is slowly emerging in the pediatric cardiology literature that CRT may have an important and useful role in certain select populations with CHF. These include patients with complete heart block who develop pacing-induced cardiomyopathy, certain forms of congenital heart disease associated with systemic ventricular failure (even if the systemic ventricle is a morphologic RV) and in patients with idiopathic dilated cardiomyopathy. Studies in children supporting the use of CRT include many case reports, a few studies of CRT in post-operative patients, and one multi-center registry reporting the use of CRT in children. These papers will be summarized.

## INTRODUCTION

Cardiac asynchrony, characterized by intraventricular and interventricular conduction delay, may compromise diastolic filling, increase mitral and tricuspid valve regurgitation, worsen ventricular dilation, and impair cardiac output [1-5]. It has been long postulated that cardiac resynchronization therapy (CRT) increases the efficiency of muscular contraction and thus augments cardiac output. CRT is achieved by synchronizing the sequence of mechanical ventricular activation in patients with asynchronous ventricular contraction. CRT has now been well established as having a mortality benefit in adult patients with dyssynchronous left ventricle (LV) contraction and advanced congestive heart failure and the implantation of CRT systems is routinely applicable in these patients [6-8]. 

In children and patients with congenital heart disease (CHD), cardiac dysfunction is a major cause of morbidity and mortality. Questions remain unanswered pertaining to the actual value of CRT in this patient population. Only recently have trials started to look at the beneficial effects of cardiac resynchronization therapy in this subset of patients. 

## INDICATIONS FOR CARDIAC RESYNCHRONIZATION THERAPY IN CHILDREN

	Indications for CRT in adults include patients with New York Heart Association (NYHA) class III/IV heart failure, ejection fraction <35% and QRS duration >120ms. The strict application of these criteria in the pediatric and congenital population has many limitations. The NYHA classification criteria are designed for adults, not children. Estimation of ejection fraction in patients with complex anatomies is difficult. The morphologic right ventricle (RV) may be systemically positioned and more prone to dysfunction or only one functional yet failing ventricle may be present. The QRS duration varies with age such that there may be significant mechanical dyssynchrony despite a QRS duration <120ms in a child. Even in adults with heart failure, all patients with a ventricular conduction abnormality may not benefit from CRT and some patients with a normal QRS duration derive benefit from this therapy [9]. Electrically measured ventricular dyssynchrony by ECG may be looked at as a relatively crude reflection of mechanical ventricular dyssynchrony. Unlike their adult counterparts with heart failure where LV dyssynchrony and a left bundle branch block (LBBB) is usually observed, RV dyssynchrony and a right bundle branch block (RBBB) is much more common in children. Therefore, although there are relatively few pediatric patients who would meet the adult CRT criteria, there remains a subset of younger patients with intrinsic or acquired conduction delay who could benefit from this technology.

Based on the adult literature, children with a dilated cardiomyopathy and dyssynchronous ventricular contraction would be an ideal subset of patients to benefit from CRT. Idiopathic dilated cardiomyopathy in children has a high rate of mortality. In patients with severe dilated cardiomyopathy who fail to respond to conventional medical therapy, CRT may prevent or prolong a cardiac transplant in some patients. Surgical repair may also contribute to ventricular asynchrony.

Patients with single ventricle physiology are at risk of developing heart failure, associated with increased mortality. Myocardial dysfunction remains the leading cause of death after stage I Norwood palliation and following the Fontan procedure [10-12]. In a subcohort of patients with single ventricles followed for a median of 17 years post Fontan palliation, 40% developed congestive heart failure and 18% died [13].

Complete heart block, both congenital and acquired, is the single most common indication for pacing in children. These patients are routinely paced from the RV. Evidence is mounting that conventional pacing in the RV might have detrimental effects [14-16]. The Dual Chamber and VVI Implantable Defibrillator (DAVID), the Mode Selection Trial in Sinus-Node Function (MOST), and other trials have shown that right ventricular (RV) apical pacing has deleterious effects on LV function, most likely as a result of inducing LV dysynchrony [14,16,17]. Kim *et al. *reported the incidence of left ventricular dysfunction in patients with congenital complete AV block in those who have been paced for more than 10 years to be around 10% [18]. Thus in patients with complete heart block, it may be important to restore not only appropriate AV synchrony but also to preserve the process of cardiac mechanical activation.

## REVIEW OF LITERATURE

### Adult Literature

The first case report introducing the concept of CRT for treatment of congestive heart failure was published in 1994 [19]. CRT in the management of CHF was subsequently validated in two randomized adult trials. MUSTIC [20], the first trial, compared in a single-blind, 3 × 3 months crossover design active versus inactive biventricular stimulation in a group of patients in sinus rhythm and another group in atrial fibrillation. Both phases of the trial demonstrated significant positive effects conferred by CRT. The number of hospitalizations for management of CHF was decreased by 2/3, and 85% of patients preferred the atrio-biventricular over the inactive stimulation mode. These results were amply confirmed by the MIRACLE trial [21] which showed reverse remodeling with CRT and the COMPANION trial [22] which found that in patients with advanced heart failure and a prolonged QRS interval, cardiac-resynchronization therapy decreased the combined risk of death from any cause or first hospitalization and, when combined with an implantable defibrillator, significantly reduced mortality.

### Acute Postoperative CRT

Initial studies in children and patients with CHD used temporary stimulation in the post-operative period to assess the benefits of CRT. Janousek *et al. *[23] showed that CRT increased systolic blood pressure in post-operative pediatric patients with biventricular repairs and intraventricular conduction delay. This group found that an increase in blood pressure positively correlated with initial QRS duration and extent of QRS shortening. Zimmerman *et al. *[24] examined the effect of resynchronization therapy on the post-operative pediatric patient after surgical repair. This group also found improvement in systolic blood pressure and cardiac index and a decrease in QRS duration. Pham *et al. *[25] recently reported results at odds with previously discussed studies. They found no change in systolic blood pressure either with conventional dual-chamber pacing or biventricular pacing when compared with baseline conditions. However, unlike the three aforementioned studies, this study did not have significant baseline intraventricular conduction delay (mean QRS duration of 95 ± 18 ms *vs*. a median QRS duration of 120 ms in the Janousek *et al. *study). It is possible that biventricular pacing did not improve blood pressure in these patients as they may have been too well synchronized at baseline. 

### CRT in Right Bundle Branch Block

Prolonged QRS duration is a marker of increased sudden death risk in tetralogy of Fallot. RV pacing decreases QRS duration, potentially offering RV resynchronization in patients with baseline RBBB. This form of CRT is by RV and not Bi-ventricular pacing.

Stephenson *et al. *explored electrical resynchronization by RV pacing and AV interval optimization in 28 patients with tetralogy of Fallot and RBBB [26]. Overall, QRS duration was significantly shorter with right ventricular pacing compared to unpaced rhythms (140 ± 30 ms versus 170 ± 20 ms). Hemodynamics were, however, not assessed and the clinical significance of these findings was not determined.

Electrical resynchronization was further assessed in 7 patients with RV dysfunction and RBBB by Dubin *et al. *[27]. Transvenous pacing catheters were positioned in the right atrium and RV. The AV interval was programmed to 90% of the PR interval. RV pacing sites included apex, outflow tract, and septum. Overall, sequential AV pacing improved cardiac output and RV dP/dt and decreased QRS duration when compared to atrial pacing alone and normal sinus rhythm. There was a strong relationship between degree of QRS improvement and increase in cardiac output.

### CRT in Systemic Right Ventricle

In congenital heart disease with two functional ventricles, the RV is systemic in congenitally corrected (L-TGA) and complete (D-TGA) transposition of the great arteries with intraatrial baffle repair. Although arterial switch surgery has supplanted atrial correction as the procedure of choice for D-TGA, thereby restoring the LV to its systemic position, the large majority of adults with D-TGA have had atrial switch procedures. Over one third develop moderate to severely depressed systemic right ventricular function and impaired exercise tolerance [28]. In a large cohort, clinical heart failure was recognized in 22% with D-TGA and intraatrial baffles and in 32% with L-TGA [29]. The systemic ventricular ejection fraction was significantly lower in symptomatic (35 ± 16%) compared to asymptomatic (47 ± 13%) patients. Moreover, at 15.7 years of follow-up, mortality was 47% in symptomatic patients compared to 5% in asymptomatic patients.

CRT for systemic right ventricular dysfunction was first reported in a 24 year-old man with L-TGA, ventricular septal defect, and pulmonary atresia with NYHA class III symptoms and severe biventricular dysfunction [30]. Transvenous CRT resulted in improvement of symptoms to NYHA class I, decrease in ventricular dimensions, and improvement in fractional shortening within 1 month of institution.

Janousek *et al. *assessed the hemodynamic benefits of CRT in 8 patients with systemic RVs [31]. Two had RBBB and 6 had left ventricular pacing-induced conduction delay with a QRS interval of 161 ± 21 ms. Six had CRT for systemic right ventricular dysfunction despite standard medical therapy and 2 received CRT as a preventive measure during thoracotomy for another indication. Change from baseline rhythm to CRT was accompanied by a decrease in QRS duration, reduction in interventricular mechanical delay, and immediate improvement in right ventricular filling time, Tei index (isovolumic relaxation time plus isovolumic contraction time divided by ejection time), maximum right ventricular dP/dt, and aortic velocity time integral. Right ventricular ejection fraction assessed by radionuclide ventriculography increased by 10% on average. On follow-up, right ventricular fractional area of change increased from 18% prior to resynchronization to 30% at 17.5 months. 

### Single Ventricle

The objective of CRT in single ventricles is not biventricular but intraventricular resynchronization through multisite pacing. Techniques such as 3D echocardiography are proving useful in assessing synchronization of individual contracting segments in patients with single ventricles [32,33]. Multisite pacing was assessed in the acute postoperative setting in 26 patients, with single ventricles undergoing some form of surgical palliation [34]. With multisite pacing, QRS duration decreased in 24 of 26 patients (94 versus 72 ms), systolic blood pressure increased in 25 of 26 patients (86 versus 94 mmHg), cardiac index increased in 21 of 22 patients (3.2 versus 3.7 L/min/m 2 versus), and 3D echocardiographic index of synchrony improved in 8 of 10 patients (10.3 *vs*. 6.0). Senzaki *et al. *described a case report of an 18 year old patient with a single ventricle who was repeatedly hospitalized for heart failure and cyanosis and deemed inoperable [35]. Institution of CRT resulted in improvement in NYHA from class IV to II and ejection fraction from 20% to 45%.

### Permanent CRT

Because CRT has only been recently instituted in this patient population, there is limited data on its long term benefits. Strieper *et al. *[36] describe the early clinical benefits of CRT in a select group of seven pediatric patients with severe congestive heart failure symptoms and congenital heart disease referred for consideration of cardiac transplantation for refractory ventricular dysfunction. Epicardial and endocardial left ventricular leads were implanted in 4 and 3 patients, respectively. Epicardial systems were used as an upgrade to existing epicardial leads or when smaller patient size precluded introducing transvenous leads. At a median of 19 months, 1 patient died, 1 had cardiac transplantation, and 5 were removed from transplant consideration because of symptomatic improvement. These patients experienced a narrowing of QRS duration, decreased LV end-systolic and end-diastolic dimensions, and an improvement in LV ejection fraction from 16% to 36%. Four of 5 patients with improvement in clinical status had previously undergone pacing for acquired or congenital AV block. 

Dubin *et al. *[37], in a multicenter study with 22 institutions, evaluated the short-term (Median duration of follow-up: 4 months, range 22 days to 1 year) safety and efficacy of CRT in 103 children and CHD patients. CRT resulted in a significant shortening of the QRS duration and improvement in systemic ventricular ejection fraction. Of 18 patients who underwent CRT while listed for heart transplantation, 3 improved sufficiently to allow removal from the transplant waiting list. They concluded that although this population differed substantially from the adult population, CRT appeared to offer substaintial benefit. Moak *et al. *[38] sought to demonstrate the benefits of CRT on improvement in cardiac function and clinical outcome in young patients that developed congestive heart failure (CHF) and DCM following cardiac pacing for AV block. They reviewed six patients who developed CHF or low cardiac output symptoms and DCM following implantation of right ventricular (RV)-based pacing systems for AV block, and subsequently underwent CRT. CRT proved benefitial for young patients that developed CHF and DCM following RV pacing for AV block. Based on their results, they suggested early upgrading to biventricular pacing in the management of these patients. Janousek *et al. *reported two cases of children with complete heart block with a dual-chamber right ventricular-based pacemaker that developed dilated cardiomyopathy with severe septal to left ventricular free-wall dyssynchrony . Within 4 weeks of biventricular pacing, both children showed significant improvement in left ventricular function along with reverse remodeling [39]. Khairy *et al. *[40] evaluated the effects of CRT in 13 children with CHD over an average of 16.5 ± 10.1 months. They demonstrated hemodynamic improvement with an improvement in ejection fraction and dP/dt over time.

### Technological Considerations 

Children and patients with congenital heart disease comprise a heterogeneous patient population with technical limitations for CRT device implantation. Patient size, vascular access issues, and unique forms of ventricular asynchrony make the selection of potential beneficiaries challenging. Percutaneous, transvenous implantation of the leads may be difficult or impossible in patients with complex anatomies. The other option is to implant LV leads for CRT epicardially *via *a thoracotomy. This is a cumbersome and complicated procedure especially for patients with scar tissue from previous surgeries. Reports have described some novel approaches to implanting leads in patients with CHD [41,42]. Innovative tools such as noninvasive imaging of cardiac venous anatomy with 64-slice multi-slice computed tomography and noninvasive assessment of left ventricular dyssynchrony by 3-dimensional tissue synchronization imaging in patients with heart failure scheduled for cardiac resynchronization therapy may result in acute improvement of LV dyssynchrony and systolic function after CRT implantation [43].

## CONCLUSION

Although there are no prospective and randomized trial data, retrospective series show that CRT is similarly effective for managing dyssynchrony-associated heart failure in this younger population as it is for treating adults with ischemic and idiopathic dilated cardiomyopathy. Cardiac resynchronization therapy, although offering significant benefit to some patients, is clearly not for everybody. As is the case for many medical innovations, the details of patient selection are key to the successful deployment of this new treatment modality. Further work is necessary to delineate, in this complex and heterogenous group of patients, who will benefit and who will not. 
